# Bacterial Diversity and Succession in the Presence of Steel and Effects on Corrosion

**DOI:** 10.1111/1758-2229.70119

**Published:** 2025-06-10

**Authors:** Liam Nolan, Théo Risser, Rainier Catubig, Abhirami Venugopal, Jess Glasson, Damien L. Callahan, Anthony Somers, M. Leigh Ackland, Agnes Michalczyk

**Affiliations:** ^1^ Centre for Cellular and Molecular Biology, School of Life and Environmental Sciences Deakin University Burwood Victoria Australia; ^2^ Institute for Frontier Materials Deakin University Burwood Victoria Australia

**Keywords:** bacterial cultures, biofilm, carbon steel, microbial influenced corrosion, operational taxonomic unit

## Abstract

Steel corrosion is an extensive problem worldwide, substantially impacting marine infrastructures. In this study, the influence of steel on bacterial succession and corrosion was investigated by culturing marine water samples with and without steel coupons for 14 days. Compared to abiotic controls, oxygen levels were rapidly depleted in biotic cultures. Fe levels increased in controls compared to biotic cultures, potentially due to anoxic conditions and the incorporation of Fe in the biofilm. Proteobacteria dominated the initial cultures, but over 14 days the number of phylogenetic groups decreased overall in abundance. Taxons that increased in abundance included *Clostridiaceae*, *Fusobacteriaceae*, *Flavobacteriaceae* and *Prolixibacteraceae*, some members of which can utilise Fe. While initially in low abundance, *Arcobacteraceae*, *Pseudoalteromonadaceae*, *Rhodobacteraceae* and *Rhizobiaceae* numbers increased over time. Sites 1 and 2 cultures displayed localised deep pitting corrosion on coupon surfaces, consistent with microbial action, with an increase in Bacteroidetes, suggesting this phylum facilitates corrosion. In contrast, Site 3 cultures displayed uniform, superficial corrosion, with *Clostridiaceae* being the dominating family by Day 14, suggesting corrosion inhibition through biofilm formation. By identifying bacteria associated with corrosion, targeted approaches to corrosion reduction may be developed through identifying significant metabolic pathways by transcriptomics and the application of metabolic inhibitors.

## Graphical abstract

We identified marine environmental bacteria that can stimulate or inhibit the corrosion of steel, using DNA sequencing. Surface imaging of corroded metal indicated variations in corrosion patterns and biofilm dynamics driven by distinct bacterial families over time. Identifying corrosion‐associated bacteria enables targeted strategies to mitigate steel corrosion in industrial settings.

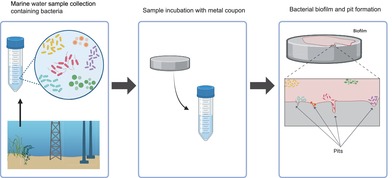

## 1 Introduction

Corrosion of steel is a global problem that affects numerous industrial sectors. In a marine environment, it causes deterioration of structures including pipelines, infrastructure, equipment and shipping vessels. Marine corrosion is estimated to cost $78 billion per year in Australia (The Australasian Corrosion Association Inc. 2020). The corrosion of iron is an electrochemical process where metal oxidation is coupled to the reduction of a suitable oxidant. Microbes affect the electrochemical reactions at the biofilm/metal interface and can inhibit or accelerate the process of metal corrosion and have been estimated to contribute to 20% of the total costs of corrosion (Koch 2017). Numerous microorganisms found in the marine environment, including fungi, archaea, algae and various bacteria, have been associated with corrosion. Many varieties of bacteria have been isolated from marine corrosion sites and are implicated in corrosion processes. The anaerobic sulphate‐reducing bacteria (SRB) belonging to the phylum Proteobacteria, the most abundant bacteria in marine environments, are reported to be the major cause of corrosion under anoxic conditions and the main mediators of the marine corrosion of iron through the release of hydrogen sulphide in the presence of sulphate (Enning and Garrelfs 2014). Members of the facultative anaerobic *Enterobacteriaceae* family (Bermont‐Bouis et al. 2007) are also associated with marine corrosion. In vitro studies show the corrosive‐promoting effect of bacteria on materials including stainless and carbon steel, where surface imaging indicated the Gammaprotobacterium *Pseudomonas aeruginosa* stimulated pit formation on duplex stainless steel (Xu et al. 2017) and 304 stainless steel (Hamzah et al. 2013) after 14 days. Pitting corrosion has been detected in cultures with the Proteobacterium *Shewanella oneidensis* after 5 days of culture with 1018 mild steel (Miller et al. 2018). However, other studies show inhibitory effects of bacteria on corrosion as observed in *Shewanella oneidensis* on 1018 mild steel (Dubiel et al. 2002), *Bacillus subtilis* on C26000 brass (Mansfeld et al. 2002), *Pseudomonas alcaligenes* and *Pseudomonas cichorii* on mild steel (Chongdar et al. 2005) and *brevibacillus* sp. on pipeline steel (AlAbbas et al., Alabbas et al. 2013).

While the corrosive characteristics of individual microbes have been identified, studies on microbial succession of environmental samples with multiple microbes on metal coupons in vitro indicate that the cultures are in a dynamic state where bacterial abundance varies over time (Moura et al. 2018; McBeth and Emerson 2016). The corrosion process is influenced by the presence of the biofilm, a dynamic aggregate of microorganisms embedded in a secreted matrix of exopolysaccharides. The biofilm enables the cooperation of microbial communities and regulates the microenvironment including the oxygen gradient and pH (Procopio 2019). Many members of the Proteobacteria phylum are biofilm generators such as the iron‐oxidising bacteria that participate in the initial colonisation of the metal surface and formation of the biofilm (Moura et al. 2018). This provides a favourable environment for the colonisation of microbial communities including anaerobic bacteria such as sulphate‐reducing, sulphur‐oxidising bacteria, iron‐reducing, iron‐oxidising bacteria, acid‐producing bacteria and others that function co‐operatively to cause corrosion (Ma et al. 2020).

Ports are regions of high marine traffic and infrastructure that generate pollution and receive contaminants from land‐based industry. Organic matter, metals and other pollutants present in the water provide an environment that supports the growth of a diverse range of bacteria (Tamburini et al. 2020) many of which may contribute to corrosion. The Port of Melbourne, a major shipping facility, is located in the estuary of the Yarra River that runs through the city of Melbourne, in Australia. The Yarra River enters Hobson's Bay, which is 3.5 km from the ocean of Port Phillip Bay. A report by the State of the Yarra and it's Parklands (2018) indicates a high level of pollution in the Yarra River, with 16 out of 25 environmental health indicators specified as poor.

The aims of this study were to measure changes in bacterial diversity and corrosion of steel coupons in samples of water cultured over time. Water samples were obtained from three locations in the Port of Melbourne. Each sample of water was placed in an in vitro culture container and bacteria in the sample were grown for 2 weeks in the presence of mild steel coupons. Water samples were analysed for bacterial diversity and phylogeny, surface metal corrosion was visualised by SEM and optical profilometry, and media Fe was measured by ICP‐MS relative to an abiotic control over time. This enabled us to identify taxa that persisted in a metal environment and that may play a key role in corrosion.

## 2 Experimental Procedures

### 2.1 Sample Collection and Preparation

**FIGURE 1:**
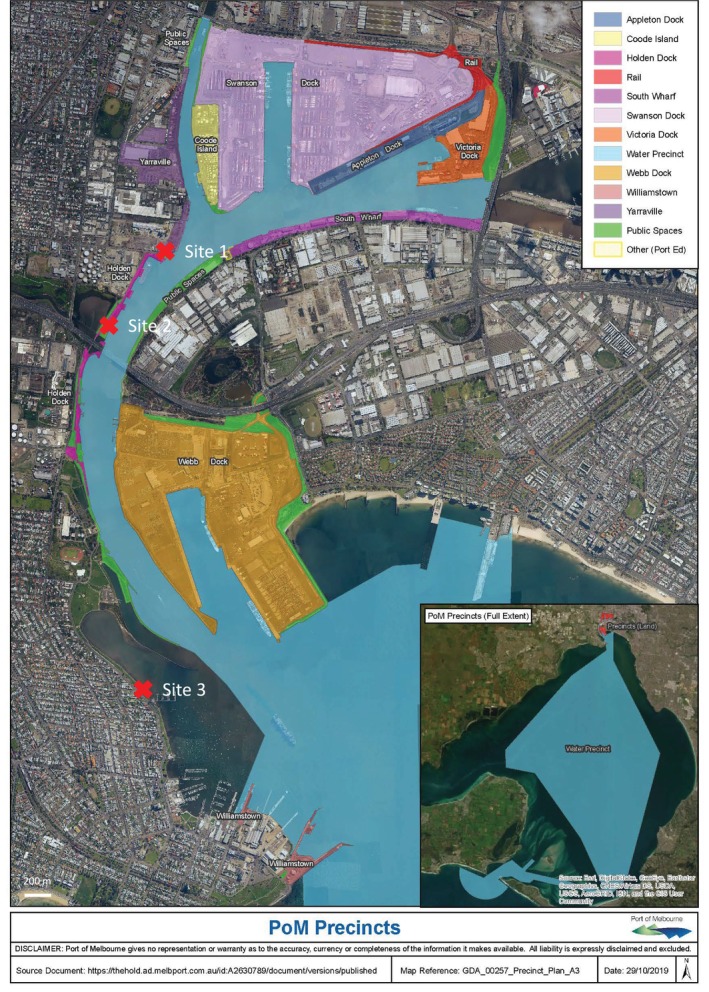
Map of Port of Melbourne location from which samples were collected at Sites 1, 2 and 3 indicated by red crosses (https://www.portofmelbourne.com/about‐us/port‐map/).

Collected water samples (100 mL) from the Yarra River were collected at three sites, Sites 1, 2 and 3 (Figure 1). The Fisherman's Bend location was chosen as it was a central area of marine infrastructure. The water samples collected at the three sites were adjacent to corroded metal structures in the marine environment and contained some silt. After collection, samples were stored at 4°C. The salinity of the field samples was 22.1, 22.5 and 24.8 g/L at Sites 1, 2 and 3, respectively, and the pH was 7.97, 8.21 and 8.68 for the three sites, respectively.

### 2.2 Culture Medium

Artificial sea water (ASW) was prepared with 26.5 g/L NaCl, 2.4 g/L MgCl_2_, 3.3 g/L MgSO_4_, 0.7 g/L KCl, and 1.1 g/L CaCl_2_, a modified version of the American Society for Testing and Materials (ASTM) International standard D1141‐98 artificial seawater (ASTM 2013). Once autoclaved at 121°C for 40 min, a media blend of Tryptone soya broth (TSB) and ASW (50:50) was prepared.

### 2.3 Bacterial Culture

Environmental water samples (0.1 mL) were inoculated into 25 mL of media blend and further diluted (1:10). Samples were incubated at 30°C, shaking at 100 rpm, with samples collected on days 1, 3, 5, 7, 10 and 14. Every 3 days, 40% of the media was replaced. Corresponding abiotic control cultures without inoculation of water were established and maintained under the same conditions.

### 2.4 Metal Coupon Preparation

AS‐1030 carbon steel coupons were obtained from a single batch of material, with composition determined by inductively coupled plasma atomic emission spectroscopy (ICP‐AES). Elements making up at least 0.2% of the total mass were recorded (98.26% Fe, 0.61% Mn, 0.29% carbon, 0.24% W, 0.21% Cr, 0.21% Si, 0.15% Cu, 0.03% Al). AS‐1030 carbon steel was cut into coupons of 12.7 mm diameter and 5 mm thickness. Coupons were imbedded in 4 mL of epoxy resin, with one metal surface exposed, and polished with P600 grit SiC paper using a grinder‐polisher (Buehler, England). Embedded coupons were sonicated in 100% acetone for 3 × 5 min cycles at 22.5 kHz, serially washed in deionised water and 80% ethanol, and sterilised in UV CL‐100 Ultraviolet Crosslinker (UVP, USA) for 90 min on each side.

### 2.5 OD Measurements

Culture media samples (200 μL) were taken for OD_600_ measurements using a spectrophotometer on days 1, 3, 5, 7, 10 and 14. The Day 0 readings for all cultures were subtracted from the subsequent readings as background.

### 2.6 Oxygen Concentration Measurements

O_2_ levels were measured with a dissolved oxygen meter (Orion Star A323, STARA3230 series).

### 2.7 ICPMS Analysis of Fe in Culture Medium

A 10 mL of media from each culture was collected days 3, 7 and 14 and processed for ICPMS based on Mathews et al. (2024). The insoluble material and supernatant were separated by centrifugation for 20 min at 6000 g. The supernatant was collected and filtered through 45 μm sterile filters. Supernatant samples were diluted to 1:100 in 3% HNO_3_ to a final volume of 10 mL. Insoluble samples were digested in 70% HNO_3_ for 30 min at 90°C and diluted to 3% HNO_3_ with deionised water to a final volume of 10 mL. Samples were then centrifuged for 20 min at 6000 g, and elemental analysis was carried out with an inductively coupled plasma–mass spectrometer (ICP–MS; NexION 350X, PerkinElmer, USA). Data analysis was carried out using Syngistix (PerkinElmer, Australia) software. Readings were normalised to internal standards (100 ppb Rh, 50 ppb Ir) and dilution factors taken into account were used to give an output in μg/L.

### 2.8 DNA Sample Collection

Biofilms were collected from samples for DNA extraction on days 3, 7 and 14. Coupons were collected and placed in 5 mL of phosphate‐buffered saline (PBS) with 0.1% Tween 20. Samples were vortexed for two minutes and then sonicated for a further minute at 22.5 kHz. The solution was collected and another 5 mL of PBS with 0.1% Tween 20 was added. Vortexing and sonication were then repeated three times. The liquid samples were then centrifuged at 48,000 g for 20 min. The supernatant was discarded, and the insoluble material was resuspended in 1 mL PBS. Samples were centrifuged again for 5 min at max speed. The supernatant was then discarded, and the pellet was stored at −80°C.

### 2.9 DNA Extraction, PCR and Amplicon Sequencing

DNA was extracted using a QIAMP DNA Stool Mini kit (Qiagen, Germany), following the manufacturer's protocol. DNA purity was measured by Nanodrop 1000 UV Spectrophotometer (Thermo Scientific, Australia). DNA concentration was determined using Qubit HS DNA assay (Invitrogen).

PCR was performed in triplicate for each sample with 2.5 ng of DNA and 1 μM of each primer in high fidelity PCR master mix (Thermofisher). Primers used included 341F (5′‐CCTACGGGAGGCAGCAG) and 785R (5′‐GAC TAC HVG GGT ATC TAA TCC), targeting the 16S V3/V4 rRNA gene, modified to incorporate the Illumina transposase sequence overhang. A total of 30 cycles of amplification were carried out as follows: 95°C for 1 min, 57°C for 30 s, 55°C for 30 s, 72°C for 30 s, and then incubated at 72°C for 3 min.

Amplified PCR products were combined and purified using AMPure XP beads (Beckman Coulter, USA) according to the manufacturer's instructions. Gel electrophoresis was carried out on a 2% agarose gel to visualise PCR products. Purified PCR products were indexed with 1 μM of unique dual index combinations using 5 indexing PCR cycles as follows: 95°C for 30 s, followed by 55°C for 30 s and 72°C for 30 s, and finally 72°C for 5 min. A 1.3% TAE agarose gel containing SYBR Safe (Invitrogen) and an Axygen gel documentation system was used to confirm indexing.

The library was diluted to 4 nM with nuclease‐free water with 10% PhiX library and denatured for 5 min with 0.2 M NaOH. This was then diluted in hybridisation buffer to create a final 8 pM library. The V3 and V4 regions of 16S rDNA were sequenced using an MiSeq system (Illumina, USA).

### 2.10 QIIME 2 Analysis

The FASTQ files of the resulting data was analysed using the Quantitative Insights Into Microbial Ecology 2 Platform (QIIME 2). Samples were first denoised using Divisive Amplicon Denoising Algorithm 2 (DADA2) and taxonomically assigned by utilising the QIIME 2 q2 feature classifier plugin in comparison to the Silva 132 database (341‐906R). Rep‐Seqs were filtered by de novo clustering at 97% identity, 3% uniqueness from another sample. Only samples with more than 120 reads per site were retained for analysis. Filtered biome data were obtained for 25 samples including 8 for Site 1, 8 for Site 2 and 9 for Site 3. These were combined by harvest days for additional filtering and a subset of data was created, filtering for Proteobacteria. QIIME2 was used to calculate Alpha diversity and generate a bar graph of the bacterial diversity.

### 2.11 Scanning Electron Microscopy

Coupons collected at timepoints of 1, 3, 5, 7, 10 and 14 days were washed 4 times in PBS (137 mM NaCl, 10 nM phosphate (PO_4_) and 2.7 mM KCl) before being fixed in 2.5% glutaraldehyde for 15 min and then serially washed in 50% ethanol and 100% ethanol. Fixed samples were air‐dried prior to being imaged with a Joel JSM‐IT300LV (Japan) scanning electron microscope. Six representative images from each coupon surface, equal to 18 images per experimental group, were collected.

### 2.12 Optical Profilometry

A corrosion product removal solution was prepared, based on ASTM G1‐03 (ASTM 2017)—designation C.3.5, containing HCl and deionised water (1:1) and 7 g/L of hexamethylenetetramine. Triplicate coupons were submerged in the washing solution for 15 min and then sonicated twice in 100% ethanol for 1 min. Optical profilometry was undertaken using an Olympus Lext OLS4100 (Japan) to measure pit number, size and depth of six fields of view for each coupon equal to 18 fields per experimental group. Representative 3D profilometry images of coupon fields were captured using Bruker Vision 64 software.

### 2.13 Statistical Analyses

Data were recorded and graphed in Microsoft Excel (2017), where values represent mean values ± standard deviation (SD) calculated from three independent replicate experiments. The statistical program IBM SPSS Statistics 25 was utilised for all statistical analyses. Statistical analyses were undertaken on normally distributed data with equal variance, which were produced from three independent replicate experiments. Multiple comparisons of means were determined by a one‐way analysis of variance (one‐way ANOVA) and Tukey's honest significant difference test performed on all data sets. All statistical analyses were tested against a *p* value of < 0.05.

## 3 Results

### 3.1 Salinity and pH of Samples From Three Sites

The salinity of the field samples (22.1, 22.5 and 24.8 g/L at Sites 1, 2 and 3, respectively) was lower than that of sea water (35 g/L) due to the locations of the sites near the junction of the Yarra River and Port Phillip Bay. Site 3, which was nearest to the sea, had the highest salinity. The pH of the field samples (7.97, 8.21 and 8.68) was similar to that of sea water (8.1).

### 3.2 Change in Turbidity of Cultures With Inocula From Three Sites Over Time

**FIGURE 2:**
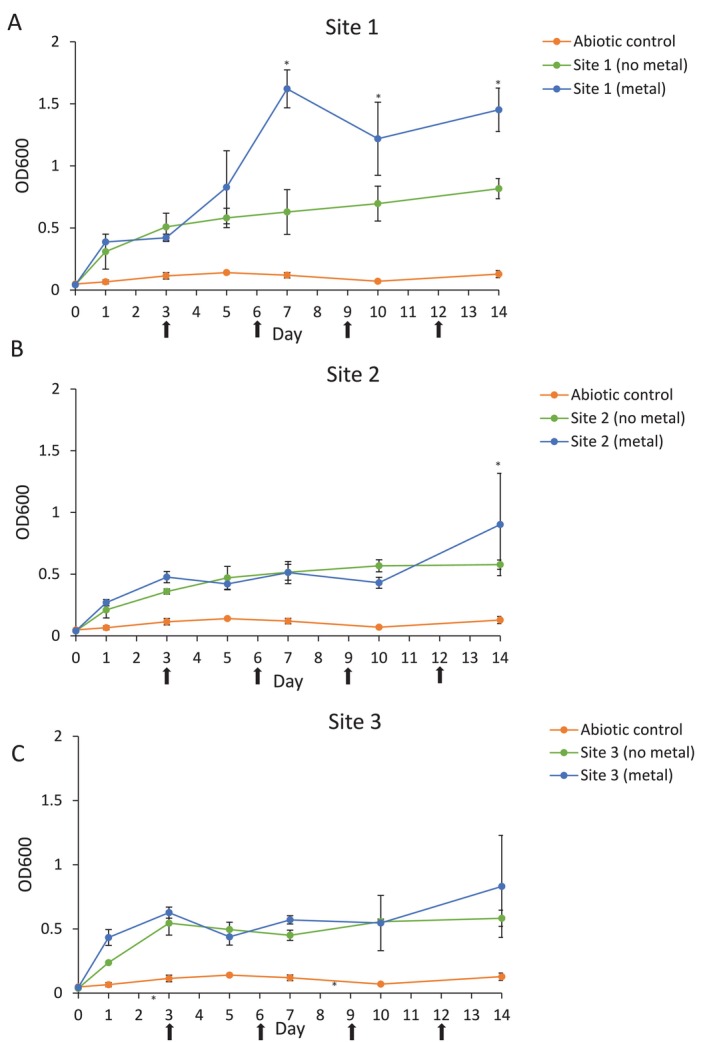
Turbidity of cultures over time. Abiotic and Site 1, 2 and 3 cultures were grown over 14 days in the presence of AS 1030 mild steel coupons. Turbidity was measured at OD_600_ on days 1, 3, 5, 7, 10 and 14. Media replacement occurred on days 3, 6, 9 and 12, as indicated by arrows. Graphs display mean and SD for three independent analyses. Significant differences (*p* < 0.05) between all groups for each day are indicated by asterisks (*).

Inocula of each of the water samples from Site 1, Site 2, and Site 3 were added to media with and without metal coupons. The abiotic control (no inocula added) showed little change in turbidity (OD_600_) over 14 days and was different from all site cultures, regardless of metal presence (Figure 2). In the absence of metal, cultures from Site 1 showed a gradual increase in turbidity, reaching a maximum reading of 0.8 (±0.1) at Day 14, while in the presence of metal, turbidity reached a maximum value of 1.6 (±0.15) at Day 7, which decreased to 1.4 (±0.18) at Day 14 (Figure 2A). Days 7, 10 and 14 exhibited a difference in turbidity dependent on metal presence. The media from Site 1 cultures changed colour over the course of 14 days, darkening over time. In the absence of metal, cultures from Sites 2 and 3 displayed maximum readings of approximately 0.5, whereas in the presence of metal, Sites 2 and 3 reached values of 0.9 (±0.41) and 0.8 (±0.40), respectively (Figure 2B,C). Sites 2 and 3 displayed a difference in turbidity between metal and non‐metal cultures on Days 10 and 7, respectively. There was no change in the colour of cultures from Sites 2 and 3 over the 14‐day period.

### 3.3 Change in Oxygen Concentration of Cultures Over Time

**FIGURE 3:**
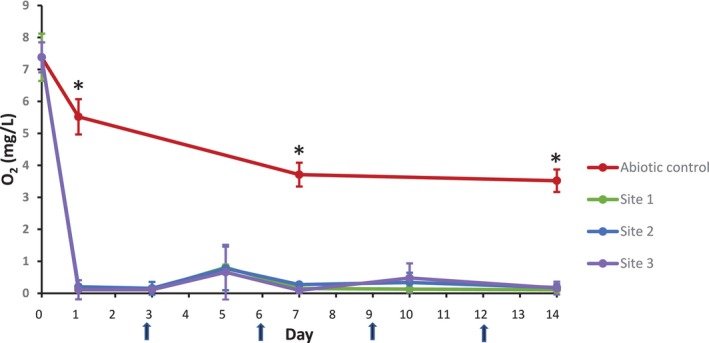
Oxygen concentration in cultures over time. Abiotic cultures and cultures containing samples from Sites 1, 2 and 3 were incubated in the presence of AS‐1030 mild steel coupons for 14 days. Media replacement occurred on days 3, 6, 9 and 12, as indicated by arrows. Graph displays mean and SD for three independent analyses. Significant differences (*p* < 0.05) between all cultures are indicated by asterisks (*).

The oxygen concentration of the media at Day 0 was approximately 7.4 mg/L (Figure 3). In the abiotic control culture, the oxygen levels reduced to 5.5 mg/L (±0.5) on Day 1 and then to 3.6 mg/L (±0.2) by Day 7, while in cultures containing microbes from all three sites the oxygen concentration fell to approximately 0.2 mg/L by Day 1 and remained similar over the 14 days of culture, with no colour change.

### 3.4 Soluble Fe in Culture Media

**FIGURE 4:**
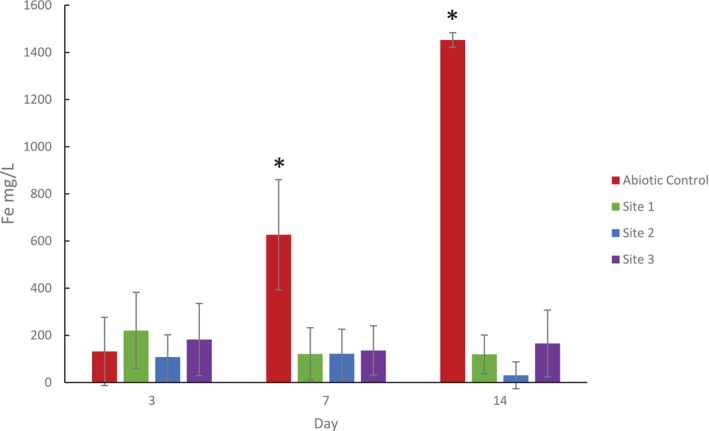
Fe concentrations in cultures over time. Abiotic cultures and cultures containing samples from Sites 1, 2, and 3 were incubated in the presence of AS‐1030 mild steel coupons for 14 days. Fe concentrations in the media supernatants were measured by ICP‐MS on days 3, 7 and 14. Graphs display mean and SD for three independent analyses. Significant differences (*p* < 0.05) between groups for each day are highlighted (*).

Fe was not present in the initial culture medium used but was detected after exposure to a metal coupon. The concentration of Fe in the culture media in the abiotic culture increased from 132 mg/L (±108) on Day 3, to 626 mg/L (±214) on Day 7 and 1453 mg/L (±47) on Day 14 (Figure 4). This was accompanied by a change in colour of the media to dark brown over 14 days. In contrast, Fe levels in cultures from all three sites were similar at approximately 100 mg/L and did not change over the 14 days of culture.

### 3.5 Scanning Electron Microscopy of the Coupon Surface

**FIGURE 5:**
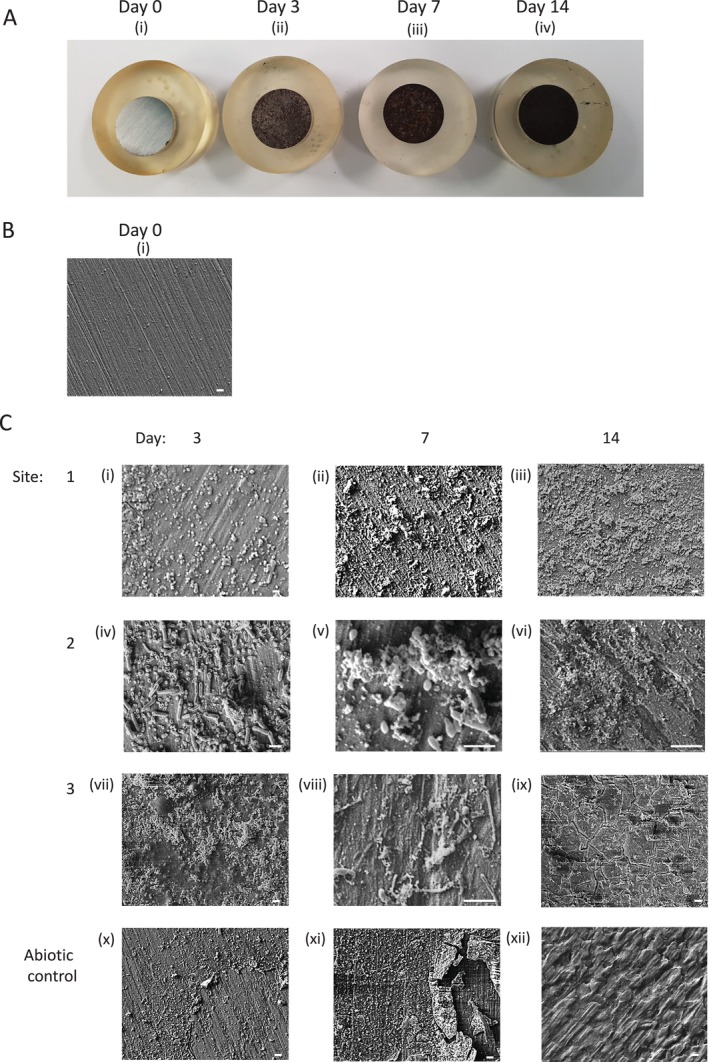
Photographs (A) and representative scanning electron micrographs (B, C) of coupon surfaces. Coupons were incubated in abiotic culture, and cultures established from samples collected from Sites 1, 2, and 3. Coupon samples were collected, biofilm was removed, processed, and coupon surfaces were imaged on Day 0 (A(i), B(i)) and days 3 (A(ii), C(i, iv, vii)), 7 (A(iii), C(ii, v, viii)) and 14 (A(iv), C(iii, vi, ix)). Abiotic controls were also imaged on days 3 (5C(x)), 7 (5C(xi)) and 14 (5C(xii)). Scale bars on the images represent 5 μm.

A progressive darkening of the coupon surface in the abiotic culture from silver at Day 0 to dark grey at Day 3 and dark brown at Day 7 and 14 was observed (Figure 5A). Under higher power, the polishing lines were clearly seen at Day 0 (Figure 5B). The abiotic control samples showed changes to the surface pattern of the coupon, including deposition of material and fragmentation of the surface at Days 3, 7 and 14 (Figure 5C(x–xii)). Cultures from all sites showed bacterial attachment and biofilm formation by Day 3 (Figure 5C(i,iv,vii)). Site 1 had the lowest density of bacteria and the lowest level of biofilm coverage from Day 3 to Day 14 (Figure 5C(i–iii)), while cultures from sites 2 and 3 showed a greater number of bacteria and higher coverage of biofilm at Day 3 (Figure 5C(iv,vii)) and by Day 14 a more extensive non‐uniform biofilm was seen in Site 2 (Figure 5C(vi)). On coupons from Site 3, cultures thicker, more uniform biofilm was detected (Figure 5C(ix,vii)). The cracks seen in this biofilm are possibly an artefact of processing produced as a result of sample shrinkage during the process of dehydration (Fischer et al. 2012).

### 3.6 Imaging of the Coupon Surface

**FIGURE 6:**
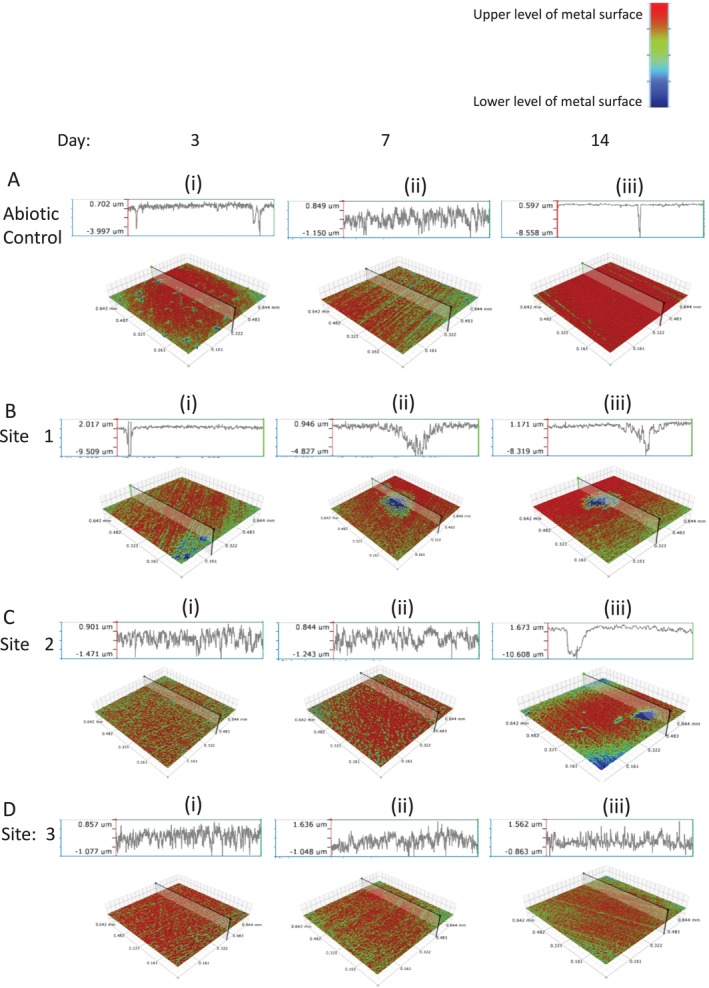
Optical profilometry of the coupon surface following biofilm removal. Abiotic cultures and cultures containing samples from Sites 1, 2 and 3 were incubated in the presence of AC1030 mild steel coupons for 14 days. Cross sectional profiles of the metal surface for the abiotic cultures at Day 3 (A(i)), Day 7 (A(ii)), and Day 14 (A(iii)) are shown. The location and depth of the pits is presented for Site 1 samples at Day 3 (B(i)), Day 7 (B(ii)) and Day 14 (B(iii)); Site 2 samples at Day 3 (C(i)), Day 7 (C(ii)) and Day 14 (C(iii)); Site 3 samples at Day 3 (D(i)), Day 7 (D(ii)) and Day 14 (D(iii)). For each sample, the highest point on the surface is indicated by red colour and the lowest point is indicated by blue colour.

Optical profilometry shows the surface of the metal coupon after biofilm removal (Figure 6). For each sample, the highest point on the surface is indicated by red colour and the lowest point is indicated by blue colour in the cross‐sectional profile. The abiotic control culture showed the presence of small, narrow pits at Day 3 (Figure 6A(i,ii)), and by Day 14 the maximum pit depth had doubled (Figure 6A(iii)). Cultures from Site 1 showed narrow deep pits at Day 3 (Figure 6B(i)), which increased in depth and volume from Day 7 to Day 14 (Figure 6B(ii,iii)). Site 2 showed small, narrow pits at Day 3 and Day 7 (Figure 6C(i,ii)) and by Day 14 the pits were wider and deeper (Figure 6C(iii)). In contrast, Site 3 showed little change from Day 7 to Day 14 (Figure 6D(i–iii)). The pit data do not indicate the amount of metal removed from the surface.

### 3.7 Bacterial Diversity and Phylogeny

DNA extracted from all initial environmental samples was processed in triplicate and analysis of the V3 and V4 regions of 16S rDNA resulted in 328,894 total reads, with a range of 16,725–77,600 reads per sample. DNA was also extracted in triplicate from 3 bacterial cultures and the abiotic control, on days 3, 7 and 14 resulting in 3,955,133 total reads (V3 and V4 region 16S rDNA), with a range of 93–300,519 reads per sample.

The median number of reads taken from each sample for analysis for the field samples was 56,241 for Site 1, 119,311 for Site 2, and 95,872 for Site 3. The number of sequences obtained from bacteria in the field samples ranged from 55,433 from the Site 1 sample, 21,773 from the Site 2 sample, and 10,281 from the Site 3 sample. In culture, the number of sequences increased with time from Day 0 to Day 14, consistent with bacterial growth. After 14 days, the Site 1 cultured sample had a 1.6‐fold increase (to 143,889) in the sequence count relative to Day 0; for the Site 2 cultured sample, there was a 10.7‐fold increase (to 255,818) in the sequence count at Day 14 relative to Day 0; and for the Site 3 cultured sample, there was an 8.3‐fold increase (to 95,133) in the sequence count at Day 14 relative to Day 0 (Table 1A–C). In the field samples, the number of OTUs (operational taxanomic units) was 168 for the Site 1 sample, 97 for the Site 2 sample, and 94 for the Site 3 sample. By Day 14, the OTUs had decreased to 51 for the Site 1 sample, 31 for the Site 2 sample, and 41 for the Site 3 sample (Table 1A–C).

**TABLE 1:** Number of sequences and alpha‐diversity indices of samples from the Port of Melbourne.

A				
Site 1				
	Day 0 (field sample)	Day 3	Day 7	Day 14
Number of sequences	55,433	48,888	57,050	143,889
Number of OTUs	168	22	58	51
Shannon index	3.8	1.3	1.8	1.8

B				
Site 2				
	Day 0 (field sample)	Day 3	Day 7	Day 14
Number of sequences	21,773	137,007	101,615	255,818
Number of OTUs	97	62	61	31
Shannon index	3.9	1.3	1.4	0.8

C				
Site 3				
	Day 0 (field sample)	Day 3	Day 7	Day 14
Number of sequences	10,281	144,548	96,611	95,133
Number of OTUs	94	54	39	41
Shannon index	4.2	1.9	1.9	2.2

The Shannon alpha‐diversity indices for the field samples were 3.8 for the Site 1 sample, 3.9 for the Site 2 sample and 4.2 for the Site 3 sample. By Day 14, the Shannon diversity index had decreased to 1.8 for the Site 1 sample, 0.8 for the Site 2 sample and 2.2 for the Site 3 sample (Table 1A–C).

The relative abundances of bacteria were determined at different taxonomic levels, from phylum to family. For the field sample, collected from sites 1, 2, and 3, the sequences obtained for each taxonomic group were between 10 and 14 phyla, 15 to 20 classes, 34 to 53 orders, and 50 to 78 families for the three sites (Table 2A–C). From the field sample to Day 14 of culture, there was an overall decrease in the abundance of taxonomic groups. For sites 1, 2 and 3, there were decreases in phyla from 14 to 8 in the Site 1 sample, 10 to 7 in the Site 2 sample, and 12 to 6 in the Site 3 sample. The number of classes decreased from 20 to 10 in Site 1 samples, 15 to 11 in Site 2 samples, and 18 to 9 in Site 3 samples from the initial field sample to Day 14 in culture. There were decreases in the number of orders from 53 to 19 in the Site 1 sample, 34 to 19 in the Site 2 sample, and 41 to 14 in the Site 3 sample after 14 days of culture. The number of families decreased from 78 to 31 in Site 1 samples, 52 to 23 in Site 2 samples, and 50 to 24 in Site 3 samples from the initial field sample to Day 14 in culture.

**TABLE 2:** Abundance of phylogenetic groups in three samples from the Port of Melbourne.

A				
Site 1				
	Day 0 (field sample)	Day 3	Day 7	Day 14
Phylum	14	7	9	8
Class	20	7	12	10
Order	53	10	25	19
Family	78	14	37	31

B				
Site 2				
	Day 0 (field sample)	Day 3	Day 7	Day 14
Phylum	10	9	8	7
Class	15	12	11	11
Order	34	21	23	19
Family	52	35	37	23

C				
Site 3				
	Day 0 (field sample)	Day 3	Day 7	Day 14
Phylum	12	9	9	6
Class	18	12	12	9
Order	41	16	17	14
Family	50	31	23	24

**FIGURE 7:**
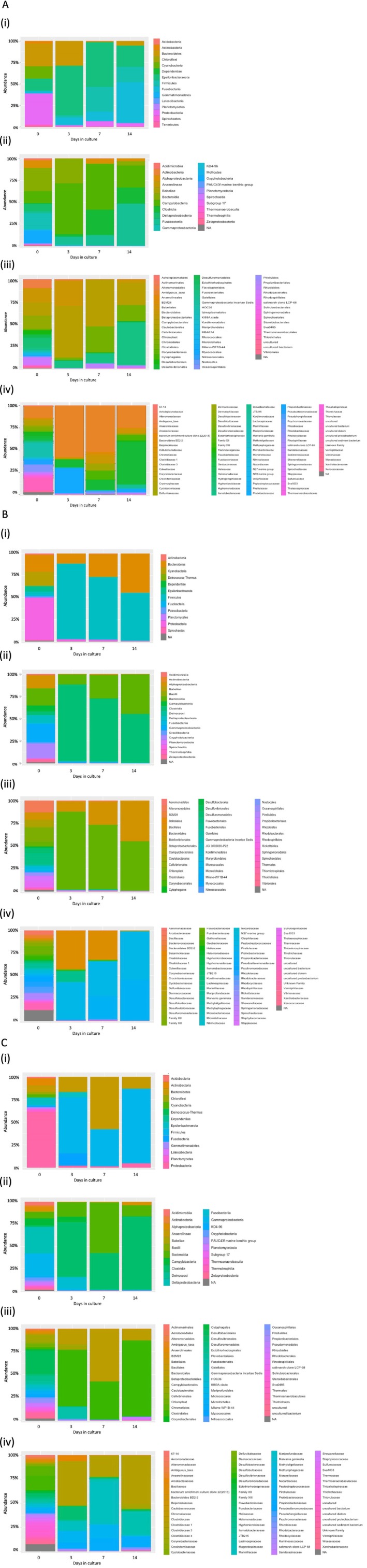
Taxonomic composition of bacteria present in samples collected from Site 1 (7A), Site 2 (7B) and Site 3 (7C) over 14 days of culturing including phylum (i), class (ii), order (iii) and family (iv) levels. Bacterial samples were collected from cultures on days 0, 3, 7 and 14, and DNA was extracted and sequenced to target the 16S V3/V4 rRNA gene fragment.

While the overall relative abundance of different taxonomic groups diminished with time in culture, some taxonomic groups increased over the 14 days in culture. For the Site 1 sample, Proteobacteria represented 36% and was the most abundant phylum in the field sample, while by Day 14 Proteobacteria represented 4% of the total abundance (Figure 7A). Fusobacteria, which represented 2% of the field sample, constituted 48% of the Day 14 culture, with Firmicutes and Epsilonbacteraeota also represented. For Site 1, Bacteroidia (28%), Gammaproteobacteria (24%) and Oxyphotobacteria (16%) were the most abundant classes in the field sample out of a total number of 20, and these diminished to 10 classes over time in culture. By Day 14, Fusobacteriia represented the bacterial majority abundance at 47%, with Clostridia (18%) and Campylobacteria (25%) also present. The number of identified orders represented in the field sample was 53, which diminished to 19 by Day 14 and was dominated by Fusobacteriales, which represented 49%, with Campylobacterales (25%) and Clostridiales (18%) also present. Site 1 field samples contained 78 different families, but by Day 14, *Fusobacteriaceae* was the dominant family, representing 47% of the population, with *Arcobacteraceae* representing 25%.

For Site 2, similar to Site 1, Proteobacteria (48%) was the dominant phylum represented in the field sample, and Gammaproteobacteria, Oxyphotobacteria, Bacteroidia and Alphaproteobacteria classes were also present (Figure 7B). By Day 14, the number of phyla was reduced to 7, with Firmicutes being the predominant phylum, representing 55%. For Site 2 cultures, the number of classes in the field sample was 15, which diminished over time in culture to 11 at Day 14, where Clostridia and Bacteroidia represented the majority at 55% and 45%, respectively. A similar pattern was found with orders, where the number of orders represented in the field sample was 34, which diminished to 19 by Day 14, where Clostridiales had a majority with 55% representation. In the field sample, Site 2 samples contained 52 different 52 families, and by Day 14, *Peptostreptococcaceae* and *Prolixibacteraceae* were the dominant families, representing 54% and 45%, respectively, of the population.

For Site 3, 12 phyla were identified, where Proteobacteria was found to be dominant and represented 61% in the field sample (Figure 7C). By Day 14, the number of phyla was reduced to 6, with Firmicutes being the predominant phylum at 83%. For Site 3 cultures, 18 classes were found in the field sample, with the majority representing Deltaproteobacteria, Gammaproteobacteria, and Campylobacteria, which diminished over time in culture to 11classes at Day 14, where Clostridia represented the majority at 83%. A similar pattern was found with orders, where the number of orders represented at Day 0 was 41, dominated by Desulfobacterales at 25%, which diminished to 19 by Day 14, where Clostridiales had a majority with 83% representation. In the field sample, Site 3 samples contained 50 different 50 families, and by Day 14, *Clostridiaceae* was the dominant family, representing over 56% of the population, with *Lachnospiraceae* and *Prolixibacteraceae* present.

**FIGURE 8:**
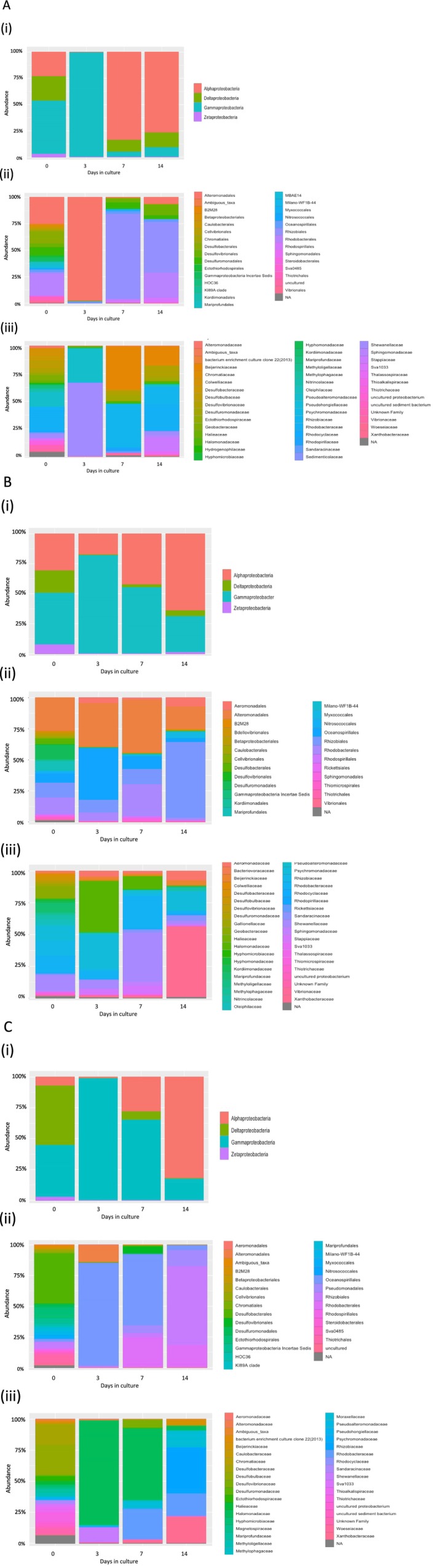
Taxonomic composition of Proteobacteria present in samples collected from Site 1 (7A), Site 2 (7B), and Site 3 (7C) over 14 days of culturing displaying abundances at class (i), order (ii) and family (iii) levels. Bacterial samples were collected from cultures on days 0, 3, 7 and 14, and DNA was extracted and sequenced to target the 16S V3/V4 rRNA gene fragment.

Due to the significant role of Proteobacteria in MIC (microbially influenced corrosion), a separate analysis was carried out on members of this phyla. After cultivation, the proportion of Proteobacteria dropped significantly in cultures from all sites. In the field sample from Site 1, Gammaproteobacteria represented 51% of the population, with Alphaproteobacteria and Deltaproteobacteria representing 23% individually (Figure 8A). While Gammaproteobacteria dominated the culture at Day 3, Alphaproteobacteria represented 84% of the Day 7 culture and 77% of the Day 14 culture. The two most common orders in the cultures from the Site 1 field sample were Altermonadales (25%) and Rhodobacteriales (21%). Altermonadales dominated the Day 3 culture (98%), while Rhizobiales dominated the Day 7 culture, representing 81%. After 14 days of culture, Rhizobiales and Rhodobacterales were the two dominant orders at 35% and 23%. *Rhodobacteraceae* (21%) was the dominant family present in the field sample from Site 1, while *Shewanellaceae* dominated at Day 3 (67%), and at Day 7, *Beijerinckiaceae* (40%) and *Rhizobiaceae* (38%) were the most abundant families. At Day 14, *Rhodobacteraceae* was the most abundant family (23%).

For Site 2, the dominant class of Proteobacteria present in the field sample was Gammaproteobacteria (42%), Alphaproteobacteria (30%) and Deltaproteobacteria (18%) (Figure 8B). After 3 days, Gammaproteobacteria had increased to (82%) and were the dominant class. By Day 7, Gammaproteobacteria represented 55% while Alphaproteobacteria represented 42% of the classes, and at 14 days in culture, Alphaproteobacteria (65%) and Gammaproteobacteria (30%) were the dominant classes. For the Site 2 field sample, there was a spectrum of bacterial orders with Alteromonadales the most dominant at 27%, followed by Rhodobacterales (13%) and then Desulfuromonadales (12%). By Day 3, Oceanospirillales was the dominant order at 42%, and at Day 7, Alteromonadales dominated (43%). By Day 14 in culture, Rizobiales was the most dominant order present at 62% with Alteromonadales (18%). Site 2 families were diverse with 39 different families represented. *Halomonadaceae* (41%) and *Rhizobiaceae* (28%) dominated at Day 3, and at 7 days, *Shewanellaceae* (39%) and *Rhodobacteraceae* (25%) were the dominant families. After 14 days in culture, *Xanthobacteraceae* dominated 56% of the culture with *Psychromonadaceae* at 16%.

The Site 3 field sample was dominated by the class Deltaproteobacteria (48%) and Gammaproteobacteria (42%) (Figure 8C). At Day 3, Gammaproteobacteria represented 99% of the Proteobacteria and 65% at Day 7. By Day 14, Alphaproteobacteria dominated 82% of the culture with 18% Gammaproteobacteria. There were 38 orders detected in the field sample, with Desulfobacterales representing 41%. Oceanospirillales represented 85% of the orders at Day 3 and 58% by Day 7, while by Day 14, Rhizobiales was the dominant order (43%) with Rhodobacterales occupying 18% and Pseudomonadales 14%. The Site 3 field sample contained 36 families, including *Desulfobulbaceae* (24%) and *Desulfobacteracea*e (17%). At Day 3, *Halomonadaceae* represented 85% of the families and *Shewanellaceae* (12%). At Day 7, *Halomonadaceae* and *Rhodobacteraceae* represented 58% and 24% of the families, respectively. By Day 14, *Rhizobiaceae* represented 38% of the families with *Xanthobacteraceae* (20%) and *Rhodobacteraceae* (18%).

## 4 Discussion

While many studies have investigated microbial corrosion in situ in marine settings (McBeth and Emerson 2016; Enning and Garrelfs 2014; Ma et al. 2020) or in flow tanks (Ramírez et al. 2016) our laboratory setting provided a regulated in vitro environment to investigate corrosion and bacterial succession in the presence of steel coupons. The closed laboratory environment reduces system variables compared to an open system such as an ocean or a bay. Evidence of microbial corrosion was indicated by the differences in surface topography between abiotic cultures and cultures of samples taken from Sites 1, 2 and 3. Optical profilometry of the coupon surface of abiotic cultures showed the presence of narrow pits at Day 14, representing a general corrosion process, while in contrast, coupons from Sites 1 and 2 contained wide pits that indicated bacterial corrosion in addition to abiotic corrosion. Localised corrosion is a key observation of microbial influenced corrosion (MIC), as most bacteria form isolated colonies on surfaces rather than a continuous layer (Loto 2017). This was likely responsible for the wide pits observed on coupons from Site 1 and Site 2 cultures. The coupons from Site 3 bacteria cultures displayed a more uniform corrosion pattern, with fewer, more narrow and shallow pits, suggesting MIC by the presence of the thick, continuous bacterial biofilm (Zuo 2007; Pantaléon et al. 2014).

The presence of wide pits in cultures from Site 1 at Day 7 and at Day 14 and in Site 2 cultures at 14 days was consistent with the location from which the samples were collected in relation to proximity to industrial activity and the number of sequences and OTUs obtained. Site 1 was the most upstream of the three sites and adjacent to the Port of Melbourne marine docks and piers, subjected to sugar, phosphate, and gypsum discharges. The field samples for Site 1 yielded 55,433 sequences, 168 OTUs, were the most abundant site for phylogenetic groups and had the lowest taxonomic diversity. Site 2 was located adjacent to marine industries including Holden Dock and yielded 21,773 sequences, 97 OTUs and had a similar taxonomic diversity as Site 1. In contrast to sites 1 and 2, field samples from Site 3 did not develop significant pits in steel coupons by Day 14. Site 3 was located at Hobson's Bay, the mouth of the Yarra, more distant from marine and industrial activities and under the influence of sea tides that dilute the river water. It had the lowest number of OTUs (94 OTUs) but the highest taxonomic diversity relative to sites 1 and 2 field samples. The variation in taxonomic diversity is consistent with previous studies which show the composition of marine microbial communities is influenced by levels of anthropogenic pollutants (Tamburini et al. 2020) where inorganic and organic pollutants can provide a substrate for bacterial growth.

Levels of soluble Fe in the abiotic cultures increased throughout the test period, from 120 mg/L on Day 3 to 1400 mg/L at Day 14. In contrast, the soluble Fe concentrations in cultures from sites 1, 2 and 3 remained unchanged between 3 and 14 days in culture at less than 200 mg/L. This difference could be explained by several factors. These include the sequestration of Fe in the biofilm, the oxygen depletion of the culture medium due to aerobic bacterial metabolism, with subsequent reduction in oxygen‐dependent electrochemical corrosion, and a protective effect of the biofilm in restricting electrochemical oxidation of Fe at the coupon surface (Knisz et al. 2023).

### 4.1 Effect of Metal on Overall Bacterial Growth

Soluble metal released from the coupon did not impact cell growth as samples from Sites 2 and 3 grew at relatively similar rates in culture over 14 days both in the presence and absence of metal as indicated by the OD_600_ readings, suggesting that access to Fe did not facilitate or inhibit planktonic growth. Site 1 showed a different pattern, where the OD_600_ increased to 1.65 on Day 7 in the presence of metal and remained higher than for the metal‐free culture up to Day 14. It was noted that the Site 1 culture developed a dark brown colour over this period which may represent the presence of SRB that formed iron sulphides (Yorshansky et al. 2022), accounting for the higher OD_600_ readings. The presence of SRB was substantiated by the ‘rotten egg’ smell indicating sulphides present in Site 1 cultures.

### 4.2 Proteobacteria Detected in Cultures From All Sites

Proteobacteria are key mediators of MIC, including SRB that belong to the class Deltaproteobacteria and are reported to be the major contributors to anoxic corrosion (Rajala et al. 2015). Using 16 s rRNA sequencing, we determined the microbial succession over a 14‐day culture period. This provided insights into the varieties of bacteria that thrived in the presence of metal and may influence corrosion. While Proteobacteria were the most abundant phylum present in the field samples, representing 37% of Site 1, 48% of Site 2, and 61% of Site 3, this phylum markedly decreased in overall abundance over 14 days in culture. There was also a decrease in the abundance of the three other phylogenetic groups (class, order, and family) over this time, which may reflect the selective loss of Fe‐sensitive bacteria and the dominance of Fe‐tolerant bacteria. Previous taxonomic analyses on metal corrosion by marine bacteria indicate the dominance of Proteobacteria under flow‐through conditions (McBeth and Emerson 2016; Moura et al. 2018; Ramírez et al. 2016). Although there was an overall decrease in the number of Proteobacteria over time in culture, some Proteobacteria increased in abundance, with a clear difference observed in cultures from the 3 sites. For Site 1, members of the Alphaproteobacteria class, *Beijerinckiaceae* family, and members of the Deltaproteobacteria class, families *Desulfovibrionaceae* and *Desulfuromonadaceae*, as well as members of the Gammaproteobacteria class, including families *Halomonadaceae*, *Pseudoalteromonadaceae*, *Rhizobiaceae*, and *Rhodobacteraceae* were increased in abundance at Day 14. Numerous species of Deltaproteobacteria from the *Desulfovibrionaceae* family contribute to metal corrosion through a metabolic process involving the reduction of sulphate to hydrogen sulphide. The hydrogen sulphide, a corrosive compound, reacts with metal surfaces to form metal sulphides, such as iron sulphides. The metal sulphides are often more soluble than the original metal, accelerating the corrosion process (Enning and Garrelfs 2014). Additionally, this SRB contributes to the formation of biofilms on metal surfaces, creating a microenvironment that enhances localised corrosion (Pal and Lavanya 2022). The presence of these bacteria in the cultures from Site 1 can explain the appearance of wide and deep pits by Day 14 and could also contribute to changes in the colour and distinctive odour of these samples.

In Site 2 cultures, no members of the Proteobacteria class increased in abundance by Day 14. For Site 3, families from the Alphaproteobacteria class including *Beijerinckiaceae*, *Moraxellaceae*, *Mesorhizobium* and *Rhodobacteraceae* increased in numbers by Day 14. The family *Rhodobacteraceae* within Alphaproteobacteria is among the nine most widely distributed bacterial lineages in marine habitats (Simon et al. 2017). Members of this family are sulphur‐oxidising facultative heterotrophs and contribute to MIC, as they will tolerate aerobic and anoxic conditions. Gammaproteobacteria was the least abundant class of Proteobacteria found in the samples from the three sites. It includes *Shewanellaceae*, a family of IRB bacteria that reduce the Fe^3+^ to the soluble, ferrous Fe^2+^ form by utilising hydrogen and other molecules even in anoxic conditions (Fu et al. 2016). Although *Shewanellaceae* was present in the field sample, it did not proliferate under the culture's conditions of this study. These variations in Proteobacteria abundance in cultures exposed to Fe for 14 days between the three sites indicate that in a complex community of microbes, bacteria can influence the abundance of each other. Although there was no significant increase in total Proteobacteria over time, the bacterial density of a culture is not a limiting factor to the bacterial‐mediated processes such as MIC (Beech et al. 1994). As sulphate‐reducing species can cause large‐scale corrosion (Enning and Garrelfs 2014) and members of the Deltaproteobacteria and Alphaproteobacteria class that increased in abundance may be candidates for Fe corrosion, particularly evident in Site 1 cultures. A study conducted on the impact of crude oil and chemical dispersant on microbial biofilm composition also reported an increase in the abundance of *Alphaproteobacteria* across all treatments in the seawater samples (Salerno et al. 2018).

### 4.3 Other Anaerobic Bacteria Present in the Cultures

In addition to the possible toxic effects of Fe, the decrease in the number of OTUs and Shannon alpha‐diversity indices from Day 0 to Day 14 for cultures from the three sites indicating a loss of bacterial diversity over time could be accounted for by conditions being more favourable for anaerobes, including ones other than Proteobacteria. The more anoxic conditions present in the cultures over time are consistent with the increase in the proportion of *Fusobacteriaceae, Clostridiaceae*, *Peptostreptococcaceae* and *Defluviitaleaceae* families over time in the cultures as these are all obligate anaerobes. Over 14 days in culture, members of the anaerobic *Clostridiaceae* family and *Lachnospiraceae* increased. *Clostridiaceae 1* were found in greater numbers in cultures at Day 14 from Site 1. The Firmicute, *Clostridiaceae 1* can reduce iron hydroxide (García‐Balboa et al. 2010) and may have contributed to corrosion. Interestingly, by Day 14, cultures obtained from Site 3 exhibited a predominance of *Clostridiaceae 1*, *3*, and *4* families, surpassing their abundance in cultures from other sites. Members of these families are recognised for their capability to generate robust and consistent mono‐ and multi‐species biofilms (Pantaléon et al. 2014). It is possible that instead of inducing corrosion, these biofilms offer some protection against MIC; however, any less protected areas may provide sites for localised attack, causing the occurrence of narrow, superficial pits observed on the coupons from Site 3. Corrosion inhibition has been documented in a multi‐species culture of *Clostridium* sp. with *Desulfovibrio caledoniensis* (Duan et al. 2008). The increase in the *Prolixibacteraceae* family members, facultative anaerobes, at all three sites following culture may also be due to the anoxic conditions favouring their growth. *Prolixibacteriaceae* have a worldwide distribution and have been isolated from marine environments (Zhou et al. 2019). Under anaerobic conditions, *Prolixibacteraceae* can corrode metallic Fe concomitantly with nitrate reduction and have previously displayed a 6‐fold increase in dissolved iron when compared to abiotic control (Iino et al. 2015). This anaerobe was found in increased numbers in Sites 1, 2 and 3 samples after 14 days of growth, relative to the field sample, and may have had a role in corrosion detected on our coupons.

*Family XII*, an obligate anaerobe, increased in cultures from sites 1, 2 and 3 after 14 days. *Family XII* belongs to the class Clostridia and may participate in corrosion, as members of this class can metabolise thiosulphate, which is associated with an increased risk of biocorrosion in oilfield installations (Smii et al. 2015). Members of *Thermaceae* were found in the Site 2 sample at very low counts. This family includes thermophilic or slightly thermophilic bacteria, with strains that are obligately oxidative, with several growing anaerobically with alternate electron acceptors and may utilise Fe (III) as terminal electron acceptors coupled to growth (Kieft et al. 1999), while others oxidise thiosulphate to sulphate in the presence of organic carbon sources (Skirnisdottir et al. 2001). *Thermus* species have been isolated from corrosion sites (Smith et al. 2019).

*Mariprofundaceae* is a gram‐negative bacterium that grows by oxidising ferrous to ferric iron. The bacterium was isolated from iron‐rich environments and can induce pitting corrosion of stainless and carbon steel (Chen et al. 2019) and was found in low numbers in all three sites.

Small numbers of members from the genus *Microbacterium* (Class Actinobacteria) were detected in cultures from sites 1 and 2. *Microbacterium* have been isolated from a wide range of environments, including heavy metal‐contaminated sites (Learman et al. 2019). *Microbacterium* isolates from heavy metal‐contaminated sites are generally more resistant to heavy metals and harbour more genes related to metal homeostasis (Corretto et al. 2020). In vitro tests showed that many strains produce siderophores which bind with metals and may enhance metal tolerance. Small numbers of microbes belonging to the family *Propionibacteriaceae* of Fe (III)‐reducing bacteria (Zhou et al. 2018) that were detected in Site 1 samples and were more abundant at Day 14 may have caused corrosion. Likewise, *Pseudoalteromonadaceae* was also found in low numbers and increased abundance at Day 14. They were reported to induce a localised attack on duplex stainless‐steel surface (Moradi et al. 2014).

### 4.4 Aerobic Bacteria Found in Cultures

The anoxic conditions developed in cultures after 1 day may account for the drop in the abundance of aerobic bacteria, including *Flavobacteriaceae* and *Pseudoalteromonadaceae* that were present in the field sample but whose abundance decreased with culturing. In contrast, some aerobic bacteria had increased their abundance in Day 14 cultures. These included *Arcobacter* from the Site 1 samples. The explanation for this may be that the conditions were not strictly anaerobic; rather, they were anoxic and sufficient oxygen was available for them to grow. *Arcobacter* is capable of chemosynthetic sulphur oxidation (Evans et al. 2018) and its presence could be another explanation for the smell of hydrogen sulphide and black colouring of the medium.

### 4.5 Bacteria Involved in Biofilm Formation

Biofilms of secreted bacterial polymers form at the interface between solid structures and liquids and provide an environment for the growth of bacterial consortia (Nwodo et al. 2012). Biofilms created by aerobes can form an anoxic environment for SRB and other anaerobic bacteria to flourish and facilitate corrosion (Procopio 2019) or inhibit corrosion (Kip and van Veen 2015). Biofilms contain inorganic ions, and the constant, low Fe concentrations seen in the culture medium over time can be explained by the incorporation of solubilised Fe compounds into the biofilm (Rizzi et al. 2020).

Bacteria attached and were visible on the surface of the metal at 3 days. Sparse biofilm was present at this time, but by Day 14, the biofilm was dense with bacteria embedded in it. Quite distinct forms of biofilm are seen between the three different cultures. The differences in the composition of consortia from the three different sites are consistent with the different patterns of biofilm formation seen with SEM. The types of biofilms present in this experiment are small, separate biofilm patches visible in cultures of sites 1 and 2. Site 3 showed larger uniform coverage by Day 3. Proteobacteria and Bacteroidetes, both present in the starting cultures, are known to participate in the establishment of the biofilm (Procopio 2019). The formation of biofilm by a Gammaproteobacteria, *Pseudomonas aeruginosa* , has been linked to the formation of pits in steel in a marine environment after 7 days (Zhou et al. 2018). Cultures from sites 1, 2 and 3 displayed a low abundance of Gammaproteobacteria in comparison to Alphaproteobacteria and Deltaproteobacteria, suggesting this may not be the prevalent corrosion‐causing bacterium. Bacteroidetes was a dominant phylum found in the biofilm on steel surfaces, as they exhibit a remarkable capacity for surface colonisation and thriving within biofilm structures. This is not surprising, considering that their genome encodes for many constituents of the EPS (McBeth and Emerson 2016). Pinpointing the exact contribution of this group within the MIC community is complex, as they could potentially benefit from the primary products of other chemolithotrophic bacteria like IRB and SRB oxidising iron and sulphur. Bacteroidetes were identified in cultures from all sites, presenting an increase in abundance over 14 days. This is particularly evident in Site 2 cultures, which display deep pits on Day 14.

The biofilm can provide an anoxic environment for anaerobes and contain mineral deposits (Ma et al. 2020) which could account for lowering the levels of soluble Fe in the supernatant in our cultures.

Microbial inhibition of corrosion may account for the reduced depth of pits found in the Site 3 culture at Day 14, relative to the abiotic control. This may be a consequence of microbes that form a protective film (e.g., the previously mentioned *Clostridiaceae* families) or due to changes in the electrochemical conditions at the metal‐solution junction (Videla and Herrera 2005). We observed a well‐established biofilm in the Site 3 culture at Day 14, which may have had a protective effect that was not present at Day 7. Electrochemically produced ferric oxides may also provide protection against corrosion, as observed in a *Shewanella oneidensis* strain that was defective in biofilm formation (Dubiel et al. 2002).

Knowledge of the specific bacteria and the mechanisms by which they influence corrosion is critical for controlling corrosion in industrial settings. Biocides, often oxidising or non‐oxidising chemicals, are used to mitigate microbial corrosion (Jia et al. 2019) however, these are toxic to many other forms of life in the marine environment. Bacterial‐specific inhibition of corroding microbes is potentially a more environment‐friendly approach to mitigate microbial corrosion. This approach would include inhibiting bacterial metabolic pathways involved in iron metabolism and biofilm formation.

## 5 Conclusion and Implications

We used a controlled laboratory setting to investigate the influence of steel on bacterial succession and corrosion in water samples collected from three polluted marine environments. Differences in surface topography between abiotic cultures and cultures from sites 1, 2, and 3 indicated microbial influenced corrosion, with varying patterns observed at different sites, potentially influenced by the proximity of the initial field samples to industrial activities and changes over time in cultures. Site 1 and Site 2 cultures exhibited localised corrosion with deep pits and trenches, which may have been formed underneath a biofilm. In contrast, Site 3 displayed a more uniform corrosion pattern with shallow surface damage. Taxonomic analyses revealed shifts in bacterial communities over 14 days, with Proteobacteria dominating initially but decreasing over time. The presence of other bacterial families representing phyla, Bacteroidetes and Firmicutes fluctuated over time and between sites, suggesting diverse microbial contributions to corrosion processes. *Clostridiaceae* families were more abundant in Site 3 cultures at Day 14, possibly contributing to corrosion inhibition through biofilm formation. Microbial inhibition of corrosion was evident in Site 3 cultures, where protective biofilms may mitigate corrosion effects, highlighting the complex interplay between microbial communities and corrosion dynamics. The results show profound changes in bacterial diversity over time in the presence of steel that may relate to their capacity to interact with Fe to cause or inhibit corrosion.

Identification of the specific bacteria associated with corrosion paves the way for a more targeted approach to reducing corrosion. Transcriptomic analysis of the bacterial metabolic pathways mediating corrosion would enable metabolic inhibitors to be selectively used to switch off bacterial biochemical events leading to corrosion. This approach would eliminate the need for non‐selective bactericide agents that are toxic to many other organisms in the marine environment.

## Author Contributions

**Liam Nolan:** investigation, methodology, writing – original draft. **Théo Risser:** investigation, methodology. **Rainier Catubig:** investigation, supervision, writing – review and editing, methodology. **Abhirami Venugopal:** investigation, writing – review and editing, formal analysis. **Jess Glasson:** investigation, writing – review and editing, formal analysis. **Damien L. Callahan:** investigation. **Anthony Somers:** writing – review and editing, supervision. **M. Leigh Ackland:** conceptualization, funding acquisition, writing – original draft, formal analysis, supervision. **Agnes Michalczyk:** conceptualization, investigation, methodology, supervision, writing – review and editing, formal analysis.

## Conflicts of Interest

The authors declare no conflicts of interest.

## Data Availability

The data that support the findings of this study are available from the corresponding author upon reasonable request.
